# On the Dynamics of the Spontaneous Activity in Neuronal Networks

**DOI:** 10.1371/journal.pone.0000439

**Published:** 2007-05-09

**Authors:** Alberto Mazzoni, Frédéric D. Broccard, Elizabeth Garcia-Perez, Paolo Bonifazi, Maria Elisabetta Ruaro, Vincent Torre

**Affiliations:** International School for Advanced Studies, Trieste, Italy; Indiana University, United States of America

## Abstract

Most neuronal networks, even in the absence of external stimuli, produce spontaneous bursts of spikes separated by periods of reduced activity. The origin and functional role of these neuronal events are still unclear. The present work shows that the spontaneous activity of two very different networks, intact leech ganglia and dissociated cultures of rat hippocampal neurons, share several features. Indeed, in both networks: i) the inter-spike intervals distribution of the spontaneous firing of single neurons is either regular or periodic or bursting, with the fraction of bursting neurons depending on the network activity; ii) bursts of spontaneous spikes have the same broad distributions of size and duration; iii) the degree of correlated activity increases with the bin width, and the power spectrum of the network firing rate has a 1/f behavior at low frequencies, indicating the existence of long-range temporal correlations; iv) the activity of excitatory synaptic pathways mediated by NMDA receptors is necessary for the onset of the long-range correlations and for the presence of large bursts; v) blockage of inhibitory synaptic pathways mediated by GABA_A_ receptors causes instead an increase in the correlation among neurons and leads to a burst distribution composed only of very small and very large bursts. These results suggest that the spontaneous electrical activity in neuronal networks with different architectures and functions can have very similar properties and common dynamics.

## Introduction

The spontaneous firing of spikes accounts for almost 80% of the metabolic energy consumed by the brain [1] and therefore this spontaneous electrical activity is expected to have a major neurobiological function. In vertebrate and invertebrate species, spontaneous activity in the nervous system has been studied primarily in rhythm-generating networks called central pattern generators (CPG) [2]. Some patterns of periodic spontaneous activity, like those underlying heartbeat [3] and respiration [4] are active throughout life, even if their frequency can be modulated. Other CPGs, like those responsible for locomotion [5], are triggered by signals coming from the environment, and are then able to sustain a periodic activity without further inputs [6]. Periodic spontaneous bursts of spikes can convey information about sensory stimuli [7] and are important also during development [8], since they contribute to determine the structure of neuronal networks [9], [10].

Neuronal networks, however, often display irregular spontaneous activity characterized by intermittent bursts separated by periods of reduced activity. From the point of view of information processing, arrhythmic spontaneous bursts represent the noise of the system under investigation [11] and it is important to determine their statistical properties. *In vivo* studies have revealed that evoked activity in stimuli driven experiments is always superimposed on this ongoing background activity, which contributes to the trial-to-trial variability [12], [13]. Most importantly, the spontaneous background activity displays spatial and temporal correlation [14], [15]. Long-range correlations of the irregular spontaneous activity have been studied in *in vitro* neuronal networks [16].

In order to identify general dynamical properties of the arrhythmic spontaneous firing, we analyzed and compared the spontaneous electrical activity of two very different networks: intact leech ganglia and dissociated cultures from hippocampal rat neurons. In the present manuscript we show that despite their differences in origin and function, the spontaneous activity of both networks has similar statistical properties. The firing of neurons exhibits a strong correlation for bin widths of hundreds of milliseconds, corresponding to low frequency correlations. Burst size and duration follow power law distributions with the same slope in both preparations.

As the dynamics of the spontaneous activity is shaped by the balance between excitation and inhibition [17] and its alteration underlies a transition between diverse bursting regimes [18], [19], we have modified this balance by means of pharmacological manipulations.

When excitatory synaptic pathways mediated by NMDA receptors are blocked, both neuronal networks are driven into a regime where large bursts are absent. In contrast, blockage of inhibitory synaptic pathways mediated by GABA_A_ receptors drives both networks into a regime characterized by large and highly synchronous bursts.

These results show that the spontaneous electrical activity of intact leech ganglia and hippocampal cultures share several properties and respond to pharmacological manipulations with very similar dynamical changes. These results also provide the background for understanding the origin and functional role of spontaneous bursting.

## Results

**Figure 1:**
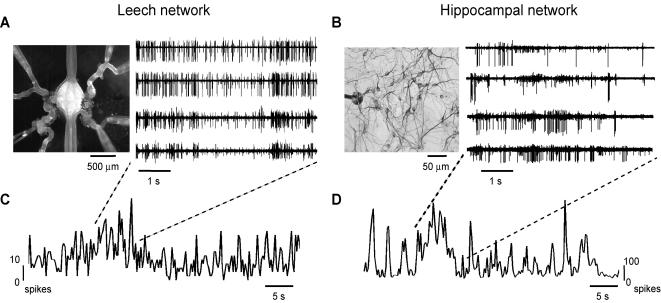
Spontaneous bursting activity in the leech and hippocampal networks. (A) Left: isolated leech ganglion with eight suction pipettes recording from different roots. Right: Extracellular recordings showing the spontaneous electrical activity monitored from four different roots. (B) Left: close-up of dissociated hippocampal neurons grown on MEA. The black dot corresponds to an individual electrode. Right: spontaneous activity recorded from four extracellular electrodes (out of 60). (C–D) Network firing rate binned at 500 ms for a representative leech network (C) and a representative hippocampal network (D). Note the presence of large peaks corresponding to concerted bursts of electrical activity and the difference between the two spike scales.

We investigated the spontaneous firing of spikes in the leech nervous system and in dissociated cultures of rat hippocampal neurons. Leech ganglia were isolated and 6 to 8 emerging roots were dissected and inserted into suction pipettes, from which extracellular voltage recordings were obtained (Figure 1A, left, see Methods). Dissociated neuronal cultures from neonatal hippocampal neurons were grown [20] on 60-channel multielectrode arrays (MEA) (Figure 1B, left, see Methods) with 50 to 55 active sites. We will hereafter refer to leech ganglia and hippocampal neuronal cultures as leech and hippocampal networks, respectively. In both preparations spontaneous activity was recorded for periods that ranged from 30 minutes to 2 hours. The firing pattern in both networks was characterized by intermittent bursts of activity with a broad variety of sizes separated by episodes of low firing activity (Figure 1A and 1B, right).

The network firing rate (see Methods) fluctuated significantly, showing large peaks corresponding to bursts of concerted electrical activity (Figure 1C and 1D). Synchronous firing was observed also in electrical recordings obtained from spatially distant extracellular electrodes of the MEA and from suction electrodes on roots emerging from opposite sides of leech ganglia.

### Network correlation at short and long time scales

**Figure 2:**
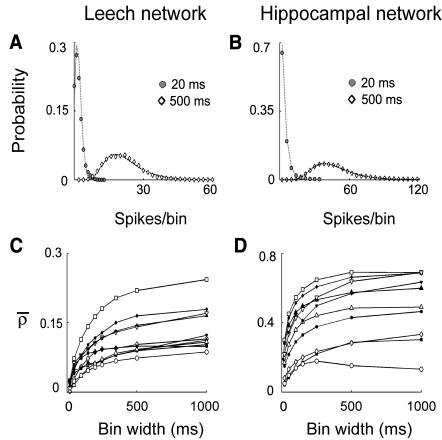
Network firing correlation. (A–B) Spikes per bin distribution of the network firing rate binned at 20 ms (filled symbols) and 500 ms (open symbols), in leech (A) and hippocampal networks (B). Data binned at 20 ms are fitted by a Poisson distribution. Data binned at 500 ms are fitted by a lognormal distribution. (C–D) Network correlation coefficient *ρ̅* as a function of the bin width for 10 leech (C) and 10 hippocampal networks (D) showing a bin width-dependent growth. Each symbol corresponds to a different preparation.

In both networks, the shape of the spike per bin distribution of the network firing rate depended on the bin width. When the activity was binned in time windows of 20 ms, the spike per bin distribution was fitted by a Poisson function in all the leech networks (n = 15, *χ* ^2^ test, p>0.05, Figure 2A), and in most hippocampal networks (7/10, *χ* ^2^ test, p>0.05, Figure 2B). In a Poisson process, leading to a Poisson distribution, the occurrence probability of a spike does not depend on the occurrence of other spikes. These results are consistent with an independent firing among neurons [21]. In contrast, when the activity was binned in time windows of 500 ms, the spike per bin distribution was fitted by a lognormal function for both networks (Figure 2A and 2B, leech, n = 15, hippocampal network, n = 10; *χ* ^2^ test p>0.05), suggesting that the networks activity is - at this time scale - regulated by interactions leading to a correlated firing [22], [23].

As different functions fitted the spike per bin distribution when different bin widths were used, the degree of correlation observed in the network could depend on the time scale in which spikes were counted. We measured the degree of correlation present in the entire network by averaging the cross-correlation at lag 0 over all pairs of neurons, thus obtaining the network correlation coefficient ρ̅ (see Methods). As shown in Figure 2C and 2D, ρ̅ grew with the bin width for most leech (14/15, r>0.5) and hippocampal networks (9/10, r>0.5). At a bin width of 20 ms, for leech networks, ρ̅ was less than 0.05 and increased by 3±1 times at a bin width of 500 ms, reaching a mean value of 0.14±0.04 (n = 15). In hippocampal networks, ρ̅ was 0.21±0.1 at a bin width of 20 ms, and similarly to leech networks, it increased by 2.5±1 times at a bin width of 500 ms reaching a mean value of 0.49±0.15 (n = 10). These results indicate that interactions leading to episodes of intense network activity take place on a timescale of hundreds of milliseconds.

### The spontaneous activity of single neurons

**Figure 3:**
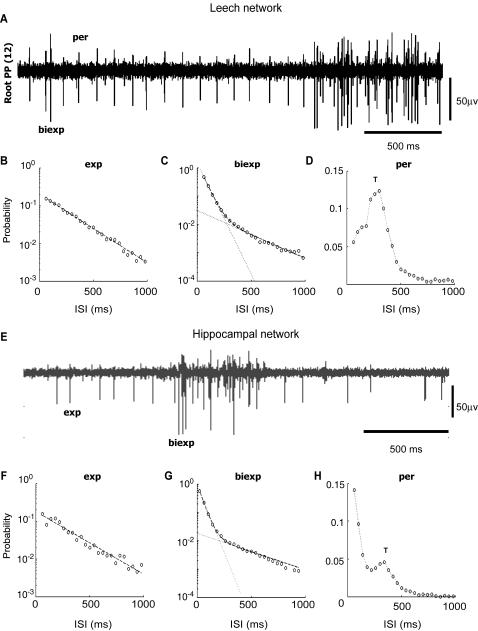
Single neurons dynamics. (A and E) Extracellular recording from a single electrode in the leech (A) and in the hippocampal network (E) showing the activity of neurons with periodic (A), Poissonian (E) and bursting (A and E) firing. (B and F) ISI distribution of identified neurons with exponential dynamics for leech (B) and hippocampal (F) network. Black dashed lines indicate exponential fit. (C and G) ISI distribution of identified neurons with bi-exponential dynamics for leech (C) and hippocampal (G) network. Black dashed lines indicate bi-exponential fit. (D and H) ISI distribution of identified neurons with periodic dynamics for leech (D) and hippocampal (G) network. Label T indicates the position of the peak, corresponding to the period of the firing.

We have studied the spontaneous activity of single neurons in both networks. From the extracellular recordings it was possible to sort spikes fired by the same single neuron (see Methods). The extracellular recordings shown in Figure 3A and 3E were obtained from the left posterior posterior root emerging from the 12th ganglion of the leech, and from a single MEA electrode, respectively. The spike sorting procedure identified in both recordings two distinct action potential shapes, one with a large and the other with an intermediate amplitude, generated by different neurons. As evident by visual inspection, in both traces one neuron fired in a regular fashion, whereas the other fired in a more irregular way, with occasional bursts of spikes.

The inter-spike intervals (ISI) of the spontaneous firing of the neurons of both networks have three stereotyped distributions: i - exponentially distributed (Figure 3B and 3F); ii - bi-exponentially distributed (Figure 3C and 3G) where the ISI probability is given by the sum of two exponential functions; iii - with a pronounced peak at a time T superimposed on either an exponential or a bi-exponential distribution (Figure 3D and 3H). The first distribution corresponds to a Poissonian firing [21], the second to a bursting neuron characterized by intervals of high frequency firing, and the last one to a neuron displaying episodes of almost periodic firing, with an average period between successive spikes equal to T. Characteristic times of each dynamics and fraction of neurons belonging to it for the two preparations are summarized in Table 1.

**Table 1:** Single neurons dynamics.

Inter spike intervals distribution	Leech network (n = 15)		Hipp. network (n = 10)	
	**char. time**	**% neurons**	**char. time**	**% neurons**
**Exponential**	175±76 ms	37% (27%–56%)	290±90 ms	19% (9%–26%)
**Bi-exponential**	42±24 ms	35% (18%–54%)	47±19 ms	70% (53%–88%)
	331±117 ms		378±194 ms	
**Periodic**	167±91 ms	27% (18%–33%)	280±74 ms	11% (0%–25%)

In the left rows for both networks characteristic times of different single neuron dynamics, i.e. time constants of exponential distributions and peak position of periodic distributions. In the right rows fraction of neurons displaying different dynamics (numbers in brackets indicate the range of the values across different experiments).

**Figure 4:**
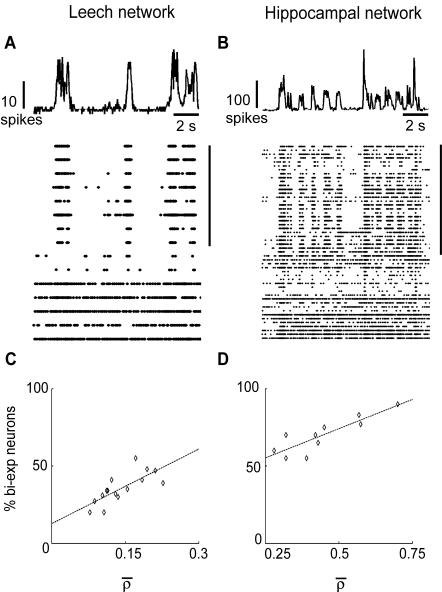
Single neurons dynamics and network bursts. (A–B) Network firing rate (upper panel) and raster plot (lower panel) for leech (A) and hippocampal (B) network. Each line of the raster plot represents the activity of a single neuron. Bi-exponential neurons have been clustered and are indicated by the black vertical bar. (C–D) Fraction of neurons displaying bi-exponential ISI distribution as a function of the network correlation coefficient for leech (C) and hippocampal networks (D). Each point represents a different experiment. Black dashed lines indicate linear regression.

Since the activity of the neurons was correlated (see Figure 2), the high firing frequency corresponding to small ISI occurred in different neurons in a quasi-synchronous way: bi-exponential neurons were bursting at similar times (see Figure 4A and 4B). Indeed, raster plots (bottom of panels 4A and 4B) clearly showed that bursts in different neurons occurred simultaneously.

It was possible to reliably identify specific leech motoneurons (motoneuron 3 and motoneuron 107, see Methods) and to analyze the variability of their spontaneous firing in different leeches. This analysis showed that leech motoneurons have a characteristic pattern of spontaneous firing, i.e. their ISI was either exponentially distributed or it had a pronounced peak indicating an almost periodic firing. However, these motoneurons could vary their behavior from a regular to a bursting firing. We then measured the percentage of neurons spontaneously firing with a bi-exponential ISI distribution across different experiments; this percentage was positively correlated to the network correlation coefficient in both leech (n = 15, r = 0.76, Figure 4C) and hippocampal network (n = 10, r = 0.85, Figure 4D), indicating that bursts were associated to an increase in correlation.

### Neuronal correlations

**Figure 5:**
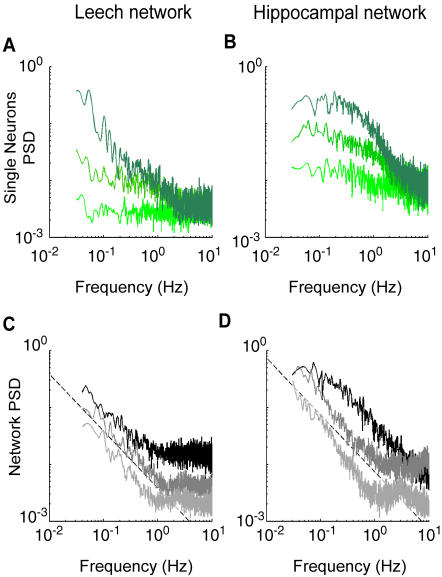
Single neurons and network frequency analysis. (A–B) Power spectral density (PSD) of representative neurons in different green shades from leech (A) and hippocampal networks (B). The PSD of the firing of a single neuron ranges from an almost flat behavior to very high power associated to low frequencies. (C–D) PSD of the network firing rate for representative experiments from leech (C) and hippocampal networks (D). Black dashed lines correspond to 1/f slope, describing the PSD for frequencies smaller than 1 Hz. In both panels, higher and lower traces are vertically shifted for clarity, by factors 1.2 and 0.8 respectively.

The three kinds of neuronal activity were associated to different autocorrelation and power spectral density functions (PSD, see Methods): i - Poisson firing neurons had an autocorrelation with a peak at zero lag, and an almost flat power spectrum (lowest traces of Figure 5A and 5B); ii - bi-exponential neurons displayed an exponential decay of the autocorrelation function with time constants ranging from 2 to 10 seconds, and an higher power associated to low frequencies, indicating that long-range temporal correlations were present during bursting activity (intermediate and highest traces of Figure 5A and 5B); iii - periodic neurons had peaks in the autocorrelation function and a single peak in the PSD, not always evident since they were superimposed on one of the two previous behaviors (data not shown).

We then analyzed the correlations of pairs of neurons in both networks (see Methods). At short time scales every neuron was correlated with only a small fraction of the network (Pearson test for independence, p>0.05). For both networks at a bin width of 20 ms every neuron was correlated with less than 10% of the network (4.6±1.5% for the leech, n = 15; 8±4% for hippocampal network, n = 10). At this time scale every neuron of the leech network was then correlated only to 1 or no other neuron, and hippocampal network neurons were correlated only to the neighboring and sometimes to a few not-neighboring neurons. The number of correlated neurons grew with the bin width, and at a time scale of 500 ms every neuron was instead correlated to approximately 60% of the neurons in both cases (57±6% for the leech, n = 15; 64±6% for hippocampal network, n = 10), indicating the presence of a coherent activity in the network. In both cases these values were not significantly different from the values obtained with a bin width of 1000 ms (60±5% for the leech, n = 15, t-test p = 0.4; 64±7% for hippocampal network, n = 10, t-test p = 0.9). Even if the extent of the spatial correlation was similar in the two networks, the synchronization was more precise among hippocampal neurons (data not shown), so the resulting network correlation coefficient was higher (Figure 2). A change in dynamics for time scales between 1 and 2 Hz was also evident as shown in the frequency analysis.

The power spectrum of the network firing rate of both networks was flat for frequencies above 2 Hz and decreased as f ^−slope^, with a slope close to 1, (leech, n = 15, mean slope = 0.97±0.3; hippocampal, n = 10, mean slope = 1.15±0.2), for frequencies ranging from 0.1 to 1 Hz (Figure 5C and 5D). This indicated long-range temporal correlations in the network activity, as a result of the structure of the activity of the single neurons and of their correlation. These results show that in both cases neurons are neither independently firing nor precisely synchronized during spontaneous activity, but that they are correlated through processes acting on the hundreds of milliseconds time scale. A bin width of 500 ms was used thereafter, since it was large enough to capture the cooperative behavior in both networks.

### Blockage of excitatory and inhibitory synaptic pathways

**Figure 6:**
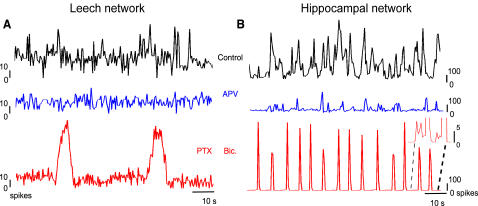
Effects of synaptic blockers on the network activity. (A–B) Changes in the network firing rates binned at 500 ms for leech (A) and hippocampal (B) networks, in control (black trace), in the presence of 20 µM APV (blue trace) and 10 µM PTX (red trace, panel A) or bicuculline (red trace, panel B). Note the residual spiking activity between periods of synchronous activity in the hippocampal network (inset, right red trace; large peaks have been truncated for clarity).

To assess more accurately the role of neurons' interactions in the spontaneous activity in both networks, we investigated the action of antagonists of excitatory and inhibitory transmission. The blockage of the excitatory transmission mediated by NMDA receptors with APV (see Methods) decreased the bursting activity (Figure 6A and 6B, blue trace) in both networks, whereas the blockage of the inhibitory pathways mediated by GABA_A_ receptors with bicuculline or PTX (see Methods) increased the bursting activity (Figure 6A and 6B, red trace), in agreement with previous results [24], [25].

It was also possible to monitor the activity of identified neurons before and after the application of receptor blockers in both preparations (see Methods) and to see that the modulation of the interactions among neurons affected the dynamics of single neurons.

**Figure 7:**
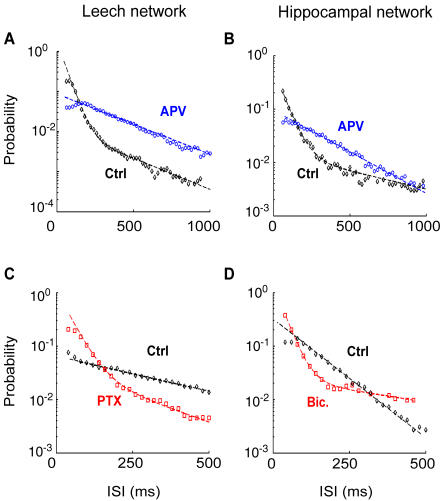
Effects of synaptic blockers on single neurons dynamics. (A–B) Neurons from leech (A) and hippocampal (B) networks, having a bi-exponential ISI distribution in control (black trace), and an exponential ISI distribution in the presence of 20 µM APV (blue trace). (C–D) Neurons from leech (C) and hippocampal (D) networks, having an exponential ISI distribution in control (black trace), and a bi-exponential ISI distribution in the presence of 10 µM PTX (red trace, panel C) or bicuculline (red trace, panel D).

In both networks the presence of APV led to an increase of the fraction of neurons displaying an exponential ISI distribution, i.e. Poissonian firing (leech: from 37% to 56% n = 6; hippocampal: from 19% to 28% n = 5). ISI distributions of neurons of both preparations switching from bursting to regular firing in presence of APV are displayed in Figure 7A and 7B. In both networks the blockage of GABA_A_ receptors had an opposite effect decreasing the fraction of neurons displaying an exponential ISI distribution (leech: from 37% to 14% n = 6; hippocampal culture: from 19% to 8% n = 5). ISI distributions of neurons switching from regular firing to bursting in both preparations are displayed in Figure 7C and 7D.

The cooperative behavior observed in both networks was investigated by studying the action of antagonists of excitatory and inhibitory transmission on the degree of correlation. We analyzed their effect on the spike per bin distribution, the power spectrum of the firing rate and the network correlation coefficient.

**Figure 8:**
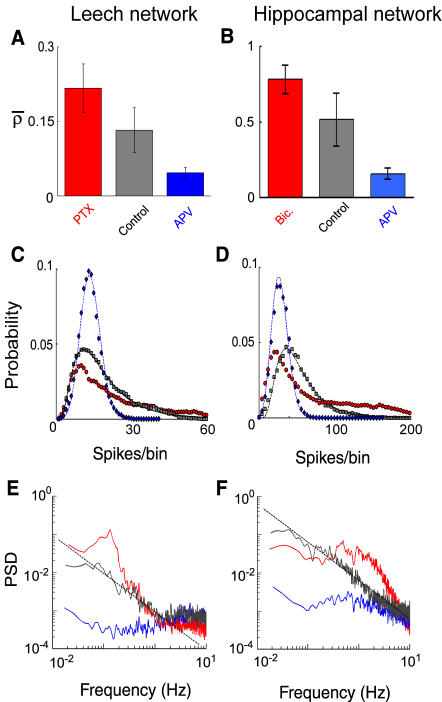
Effects of synaptic blockers on network correlation. (A–B) Network correlation coefficient rate in leech (A) and hippocampal (B) networks in the different pharmacological conditions considered. (C–D) Spikes per bin distribution of the network firing rate in leech (C) and hippocampal (D) networks. Data were fitted by a lognormal function in normal conditions (grey symbols). In the presence of 20 µM APV (blue symbols) data were fitted by a Poisson distribution for the leech network and by a lognormal distribution for the hippocampal network. Note the reduction of skewness in the presence of APV in both preparations (see text). Red symbols correspond to spike per bin distribution in the presence of 10 µM PTX (C) and bicuculline (D). (E–F) PSD of the network firing rate in control (black trace), in the presence of APV (blue trace), PTX (E, red trace) or bicuculline (F, red trace). Black dashed lines have 1/f slope.

The blockage of the excitatory transmission mediated by NMDA receptors with APV (see Methods) diminished considerably the degree of correlated firing (leech: n = 6, *ρ̅*  = 0.05±0.01, hippocampal: n = 5, *ρ̅*  = 0.14±0.03; Figure 8A and 8B). In both networks the two values were significantly smaller than in control conditions (t test, leech: p<0.05, hippocampal: p<0.05). In leech networks, the spike per bin distribution, which was fitted by a lognormal distribution in normal conditions (Figure 8C, black trace), became either a Poisson distribution (Figure 8C, blue trace; 3/6, *χ* ^2^ test, p>0.05) or a lognormal distribution (3/6, *χ* ^2^ test, p>0.05) with a reduced skewness (between 40% and 60% of the control values, see Methods) as expected from a more uniform activity with less bursts. Similarly, in hippocampal networks, the skewness of the lognormal function decreased significantly (between 5% and 20% of the control values; black and blue trace in Figure 8D).

In normal conditions, the firing rate power spectrum decreased as 1/f (Figure 8E and 8F, black traces) for frequencies between 0.1 and 1 Hz as shown above. In the presence of APV, the power spectrum was almost flat (Figure 8E and 8F, blue traces) at all analyzed frequencies (leech: n = 6, slope between 0 and −0.2; hippocampal: n = 5, slope between 0 and −0.4), as expected in the absence of correlation [26]. In contrast, when inhibitory pathways mediated by GABA_A_ receptors were blocked (see Methods), we observed an increase of correlation (leech: n = 5, *ρ̅*  = 0.22±0.05, hippocampal: n = 5, *ρ̅* = 0.7±0.1 see Figure 8A and 8B). This increase of correlation was significant in both networks (t test, leech: p<0.05, hippocampal: p<0.05) and also modified the shape of the power spectrum, leading to a broad peak at low frequencies (Figure 8E and 8F, red trace; leech, n = 5; hipp, n = 5). In the presence of bicuculline the firing rate power spectrum of hippocampal networks exhibited a pronounced peak at frequencies between 0.2 and 0.6 Hz, while PTX in leech networks increased the power associated to frequencies between 0.1 and 0.3 Hz.

These results show that excitatory synaptic pathways mediated by NMDA receptors increase firing correlation of both networks, and that inhibitory synaptic pathways mediated by GABA_A_ receptors have an opposite effect.

### Spontaneous bursting activity

Power law dynamics and long-range interactions have been found in *in vitro* cortical cultures [16], and in the propagation of spontaneous local field potentials in organotypic and acute cortical slices [27], [28]. We investigated whether the burst size and the burst duration distributions in leech and hippocampal networks also followed a power law distribution. Since there is no standard way to define a burst, we have used three different definitions (1, 2 and 3, see Methods). The definitions are based on the analysis of the ISI and on the network average firing rate. In Definition 1, the network ISI distribution is fitted with a bi-exponential function, leading to two characteristic time scales: the short time scale corresponding to the activity during the bursts, and the long time scale to the inter-burst activity. Bursts are then identified by strips of bins where the network firing rate is two times higher than the inter-burst average firing rate. Definition 2 identified bursts as collections of spikes separated by an interval smaller than the average ISI. Definition 3 identified bursts by strips of bins where the network firing rate is above its average, similarly to the procedure used in [27], [28]. Results were largely independent of the definition adopted.

**Figure 9:**
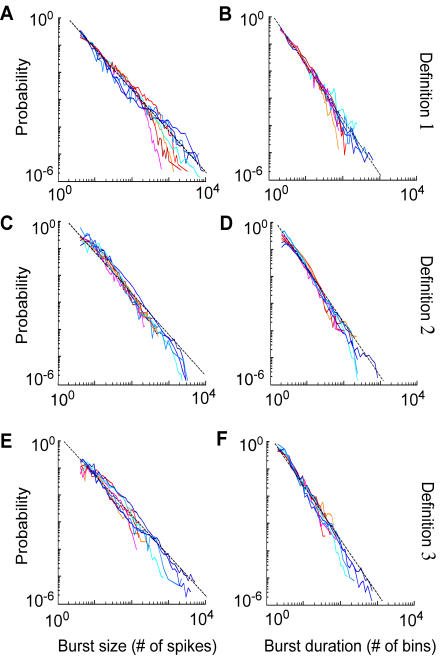
Burst statistics. Probability distributions of burst size and duration computed according to 3 different burst definitions. Data from representative experiments obtained in leech (reddish lines) and hippocampal networks (bluish lines). Bursts size (on the left) and duration (on the right) distributions are calculated according to definition 1 (A, B), definition 2 (C, D), and definition 3 (E, F). Black dashed lines are power laws with a slope of −1.5 in the left column and −2 in the right column. Note the power law behavior of bursts size distribution of hippocampal networks for 3 log units.

For leech networks and for the three burst definitions, the distributions of the bursts size and bursts duration (see shades of red in Figure 9) followed a power law over two log units. In hippocampal networks (see shades of blue in Fig. 9), where we recorded the activity of a number of neurons approximately 10 times higher than in leech networks (see Methods), we could verify power law behavior over an extended range. As shown by the blue shaded lines, the distribution of bursts size and bursts duration had a power law distribution over three log units. The average slope of the bursts size distribution (Figure 9A, 9C and 9E) was −1.6±0.2 (Def. 1), −1.55±0.2 (Def. 2), and −1.6±0.2 (Def. 3) for leech networks (n = 15) and −1.55±0.2 (Def. 1), −1.65±0.2 (Def. 2), and −1.6±0.2 (Def. 3) for hippocampal networks (n = 10). We also verified that the duration distribution of the bursts followed a power law distribution (Figure 9B, 9D and 9F) and we found an average slope of −2.1±0.2 (Def. 1), −2.2±0.2 (Def. 2), −2.1±0.2 (Def. 3) for leech networks (n = 15) and −2.1±0.1 (Def. 1), −2.15±0.2 ((Def. 2), −2.1±0.2 (Def. 3) for hippocampal networks (n = 10). The statistics of bursts obtained with the three definitions was not significantly different (ANOVA, p>0.05 for both leech and hippocampal networks). However, definition 1 was able to characterize bursts over a more extended range and it was therefore used for later analysis.

As changing the balance of excitation and inhibition significantly affected the degree of correlated firing among neurons, we analyzed how pharmacological modulations of excitation and inhibition modified the distribution of bursts size and duration.

**Figure 10:**
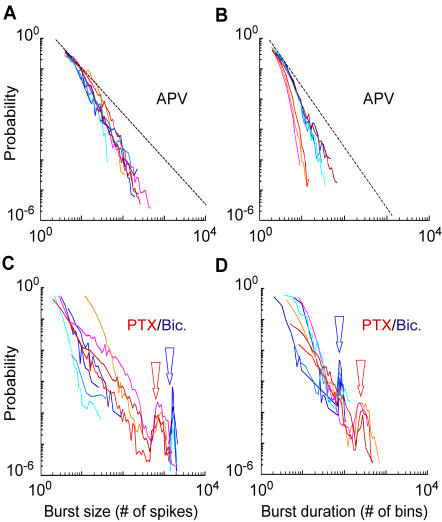
Bursts statistics in the presence of synaptic blockers. Bursts size and duration distributions for leech (reddish lines) and hippocampal (bluish lines) networks obtained using burst definition 1. (A–B) In the presence of APV, the number of large bursts decreased. The black dashed line has a slope of −1.5 in (A) and of −2 in (B), as in the right and left column of Figure 9, respectively. (C–D) In the presence of GABA_A_ receptor blockers (PTX/bicuculline), peaks corresponding to large bursts appeared (indicated by the arrows). Traces were shifted to superimpose peaks of each preparation. In hippocampal networks, intermediate size bursts are absent as shown by the discontinuity of the distributions.

In the presence of APV, distributions of bursts size and duration (Figure 10A and 10B) of leech (shades of red) and hippocampal (shades of blue) networks were concave and did not follow a power law. In contrast, in the presence of PTX or bicuculline, very large bursts became more frequent, as indicated by the presence of a peak in the burst size and duration distributions (arrows in Figure 10C and 10D). In these conditions the probability of finding bursts of intermediate size was close to zero.

These results show that when synaptic pathways mediated by NMDA receptors are blocked, and the excitatory interaction among neurons is reduced, both networks enter a regime where large bursts are absent. In contrast, blockage of inhibitory GABA_A_ synaptic pathways drives the networks towards a regime showing large and highly synchronous bursts.

## Discussion

The present study provides a detailed analysis of the spontaneous activity in two very different neuronal networks: intact leech ganglia and dissociated cultures of rat hippocampal neurons. Bursts of spontaneous spikes were observed in both neuronal networks. Despite their different origin and connectivity, several properties of spontaneous firing were present in both neuronal networks, indicating that these properties could be general features of the spontaneous activity dynamics.

### Statistical properties of the spontaneous activity

The spontaneous activity of leech and hippocampal networks, analyzed in the present manuscript, was primarily composed of irregular and arrhythmic bursts of spikes (Figure 1). These spontaneous spikes were poorly correlated when they were counted on small bin widths of tens of milliseconds. However, in both networks, the degree of correlation increased when larger bin widths of some hundreds of milliseconds were considered (Figure 2). This observation indicated the existence of temporal correlation on long time scales in both networks. Indeed the power spectrum of the network firing rate had significant components below 1 Hz, displaying a 1/f behavior in this range (Figure 5). These results are in agreement with previous analysis of cultured cortical networks [16] describing long-lasting correlation of the spontaneous activity.

The ISI distribution of the spontaneous firing of single neurons (Figure 3) was either exponential or bi-exponential or with a pronounced peak comprised between 100 and 400 ms (see Table 1). An exponentially distributed ISI was consistent with a Poisson process controlling spontaneous firing [21], whereas an ISI distribution with a pronounced peak indicated the presence of episodes of almost periodic firing. When the overall activity of the network increased, most neurons showed a bursting dynamics in which intervals of intense firing were alternated with less active periods (Figure 4 and 7). This bursting dynamics was characterized by an ISI with a bi-exponential distribution. Shifts between periodic or Poissonian firing and bursting activity produced by synaptic inputs are well known [29], and have been extensively studied in leech heartbeat interneurons [30].

The power spectra of single neuron firing could have different shapes (Figure 5A and 5B) consistent with the different ISI distributions. However, when the overall firing of the network was considered (Figure 5C and 5D) in both networks - and in all preparations - the same 1/f behavior was observed. Taken together, these observations showed that global properties of neuronal networks average specific properties of individual neurons.

The probability distributions of size and duration of bursts in both networks (see Figure 9) had a power law behavior with slopes close to −3/2 and −2 respectively.

A similar network activity dynamics is described by the Self-Organized Criticality (SOC) paradigm [31]–[33]. SOC models display long-range correlations like those of Figs 2 and 5 and when the interaction strength in SOC networks is increased or decreased, their activity distributions are very similar to the bursts distributions displayed in Figure 10. Neuronal activity was described in the SOC framework in human electroencephalogram [34], [35] (but see also [36]) and in local field potentials from cultured and acute cortical rat slices [27], but this is the first time to our knowledge that similar phenomena are found: i - at the spike level, ii - in a random neuronal network and iii - in a invertebrate network. The SOC paradigm is an interesting descriptive model for spontaneous bursts, but does not offer insights into their biological origin and into their functional role.

### Origin of the spontaneous activity

Blockage of excitatory and inhibitory synaptic pathways produced very similar effects in both networks providing some hints of the synaptic origin of the observed bursts.

Since the spontaneous bursting activity was determined by network interactions, altering the balance between excitation and inhibition caused a transition towards diverse bursting regimes. In both networks, blockage of excitatory postsynaptic potentials (EPSPs) mediated by NMDA receptors with APV reduced the degree of correlation of the spontaneous activity (Figure 8) and the occurrence of large bursts (Figure 10). Not only were networks' bursts suppressed, but also single neuron's bursts (Figure 7), supporting the finding that the single neuron behavior depends on the global correlation (Figure 4). The prominent effect of APV was unexpected in leech networks, as most of the known glutamate receptors in the leech are non-NMDA [37], [38]. However, when we applied the selective antagonist APV (see Methods) we always obtained a clear reduction of the bursting activity (Figure 6A, blue trace), suggesting that NMDA receptors are present in the leech ganglion [39]. These experimental observations indicate that excitatory synaptic pathways mediated by NMDA receptors are necessary for the correlated activity and for the presence of bursts in both networks; indeed EPSP mediated by NMDA receptors have a slow decay kinetics with a time constant of some hundreds of milliseconds [40], [41], providing the biophysical mechanism for sustained and correlated firing on longer time scales [42] (see Figure 2).

On the other hand, when inhibitory GABA_A_ synaptic pathways were blocked, a highly bursting regime appeared, with a bi-stable dynamics alternating large bursts with silent periods (Figure 6 and 10), single neurons started to burst (Figure 7) and the correlation of their activity increased (Figure 8). The spontaneous bursts became larger as excitation spread in the network more easily. In this regime, bursts duration was expected to be determined by intrinsic properties of excitatory synapses such as synaptic depletion [43], [44] and inactivation of voltage-gated currents [45]. As a consequence, the duration of the bursts became more stable (see peaks in Figure 10C and 10D).

In normal conditions, long lasting EPSP were balanced by inhibitory synaptic inputs and bursts were then likely to be terminated by inhibitory inputs that cause a propagation failure of the activity [46]: burst duration depended on the excitation/inhibition interplay in the whole network and could have a broad range of values (Figure 9). In the presence of APV, inhibition was predominant thus reducing the burst duration. Inactivation of voltage-gated currents [45] could as well influence the burst duration and time course in these two regimes.

Based on these results we propose a common mechanism for the spontaneous bursting activity of both networks: i - neurons fire spontaneously according to a Poisson process; ii - the spontaneous firing of these neurons activates long lasting EPSP, mediated by NMDA receptors, initiating a burst; iii - these bursts spread in the networks and are terminated, by the combination of several mechanisms, as discussed above. A mathematical model of these mechanisms and a comparison with experimental data is in preparation and will be presented elsewhere.

In this work we analyzed an intact and functional invertebrate network, and a dissociated mammalian neuronal network. A leech ganglion contains approximately 400 neuronal somata, as well as axonal projections from other ganglia, whereas between 10 and 100 thousand neurons form the investigated hippocampal cultures. More importantly, the leech ganglion is a hierarchical neuronal network and the hippocampal culture is a random neuronal network. In dissociated cultures, the original cytoarchitectural organization is lost during the dissociation and neurons re-wire with random connections. In contrast, leech ganglia retain a specific internal structure suitable for specific behaviors, structured for instance in mechanosensory neurons, interneurons and motoneurons. Our results showed that most features of the spontaneous activity were nonetheless shared between the two networks. This suggests that some synaptic interactions among neurons are sufficient to determine a global behavior, poorly dependent on the specific wiring of the network itself. In this view, the wiring of leech networks becomes evident when specific behaviors, such as swimming [47], [48], or crawling [49], [50] are initiated by sensory stimuli and/or command neurons [5].

The spontaneous activity in leech and hippocampal networks was not entirely similar and some features distinguished the functional leech network. The degree of correlated activity in leech networks was significantly lower than that observed in hippocampal networks (Figure 2). The dynamics of single neurons were also very different: the fraction of single neurons with a bi-exponential ISI distribution was approximately 70% in hippocampal networks and only 35% in leech networks, while the periodic neurons were 11% and 27%, respectively. Despite these differences, the similarity in the statistical properties of the spontaneous bursting activity between leech and hippocampal networks was remarkable.

### Functional role of the spontaneous activity

Spontaneous bursts play a fundamental role during neuronal development [8]. In this early phase, chloride mediated neurotransmission is excitatory [51], so the spontaneous activity is intense and in the form of long-lasting bursts alternated by periods of quiescent activity, due to the hyper excitable nature of the networks. Intense spontaneous activity is essential for the development of the functional architecture of neuronal networks [52]–[54] and for the incorporation of newly born neurons in the adult nervous system [55]. Spontaneous bursts of electrical activity of developing neurons are not only important for the refinement of neuronal projections but are also necessary for finding the correct pathways [56]. The developmental mechanisms are sensitive to activity in the time scale of several hundreds of milliseconds, and the unit of information at this stage could consequently be the burst rather than the single spike [57].

Large bursts are associated with an unblockage of NMDA receptors [58], causing a large influx of Ca^2+^ ions inside the cytoplasm [59]. This Ca^2+^ influx initiates a variety of intracellular signalling pathways, possibly also involving changes of gene expression [60], [61]. In this view, bursts of spikes have the major function of triggering fundamental biological process.

The spontaneous activity during development is also able to modulate both excitatory and inhibitory synaptic efficacy [10], thus setting the balance between inhibition and excitation present in adult neuronal networks [62]. Therefore, the interplay between growth and efficacy of neuronal connections and spontaneous activity seems to determine general features of the spontaneous activity of neuronal networks with different architectures, like those described in this work.

Global dynamical properties of the spontaneous activity of different networks can be very similar, but their neurobiological functions are expected to be different. The presence of large bursts and strong correlation impairs information processing, and indeed in dissociated hippocampal networks, a reduced spontaneous activity and a lower degree of correlation decrease response variability to the same stimulation and increase correlation between stimulus and response [63]. In intact animals large spontaneous bursts could instead have an important role acting as internal triggers in the initiation of movements, also in the absence of external stimulation [64], [65]. In fact, large bursts of spontaneous activity are statistically related to the initiation of motion in resting leeches (Garcia-Perez et al., in preparation).

As different dynamical regimes seem more suitable for different functions, it is very useful to drive a neuronal network from one regime to another. These transitions can be achieved by modifying the balance between excitation and inhibition by means of neuromodulators [66] and can also occur during development, when GABA_A_ mediated synaptic potential reverse polarity and pass from excitatory to inhibitory [67]. The spontaneous activity pattern strongly influences neuronal networks' information processing [68], and thus modulation of neuronal networks' spontaneous activity is likely to be a basic feature of neural computation [69], essential for tuning the function of neuronal networks.

## Materials and Methods

### Physiology

#### Leech preparations

Adult leeches *Hirudo medicinalis* were obtained from Ricarimpex and kept at 5°C in tap water dechlorinated by aeration for 24 hours. Leech ganglia were isolated as previously described [70] and the roots emerging from the exposed ganglion were cleaned for suction pipette recordings [71]. In each experiment we recorded from 6–8 roots the activity of 15 to 25 motoneurons. All preparations were kept in a Sylgard-coated dish at room temperature (20–24°C) and bathed in Ringer solution (in mM: 115 NaCl, 1.8 CaCl_2_, 4 KCl, 12 glucose, 10 Tris maleate buffered to pH 7.4) [70].

#### Hippocampal cultures

Hippocampal neurons were obtained from Wistar rats (P0–P2) and plated on 60-channel multielectrode array (MEA, MultiChannelSystems) enabling extracellular recordings as previously described [20], [63]. As it was possible to sort spikes from 1 to 5 neurons for an individual electrode, we were able to record the activity of a maximum of 300 neurons. The age of the cultures varied from 22 to 35 days *in vitro*. Extracellular recordings were digitized at 10 or 25 kHz, and recorded with MCRack (MultiChannelSystems, Reutlingen, Germany).

#### Pharmacological manipulations

To reduce the excitation in the two neuronal networks, we bath-applied the non-competitive NMDA receptor antagonist 2-amino-5-phosphonopentanoic acid (APV) (final concentration, 20 µM; TOCRIS). To reduce inhibition, we bath-applied the ionotropic GABA_A_ receptors antagonists picrotoxin (PTX, final concentration, 10 µM; Sigma) and bicuculline (final concentration, 10 µM; Sigma) to leech ganglia and to hippocampal dissociated cultures, respectively.

### Data Analysis

Acquired data were analyzed using MATLAB (The Mathworks,Inc.).

#### Spike sorting

Spike sorting was carried out using principal component analysis and a custom software [71] for leech networks and open source MATLAB toolboxes for the analysis of multielectrode data [72] for hippocampal networks.

In some experiments it was necessary to identify neurons to find changes in their dynamics due to pharmacological manipulation. We identified the neurons assuming that the relative action potential amplitude was not affected by these modulations. In both preparations, for each electrode, the behavior of the neurons producing the two or three largest action potentials was analyzed in control conditions and in the presence of drugs.

The position of most motoneurons in the leech ganglion is known [70]. Thus it was possible to simultaneously record intracellularly from specific motoneurons and extracellularly from the roots. Motoneurons 3 and 107 reliably produced the largest action potentials in the dorsal posterior and anterior anterior roots, respectively. Thanks to this, we could compare the activity of these identified neurons in different preparations.

#### Network firing rate

The duration of the recording was divided into bins of constant width. Different widths were used to compare properties at different time scales. For each single neuron the number of spikes occurring in each bin was counted and the resulting discrete time series represented the *neuron firing rate*. The *network firing rate* was defined as the sum of all neuron firing rates (i.e. the number of all spikes recorded in the network from each bin).

In both preparations more than one electrode can record the activity of the same neuron. In the leech, some motoneurons project their axons in more than one root. To avoid double recording, spikes separated by less than 2 ms, occurring in different roots of the same side, were counted as a single spike in the network firing rate. MEA can record the activity of a single neuron in several sites, but we estimate that this has a low probability to occurr, as the spatial density of the electrodes is low (inter-electrode distance = 500 µm).

#### Spike per bin distribution

The probability distribution *P(S)* of the number of spikes S per bin of the network firing rate was fitted either by a Poisson or by a lognormal function:
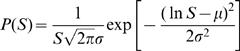
where µ and σ are the mean and the variance of the distribution of the logarithm of *S*.

#### Skewness

The skewness of a distribution, a measure of the degree of asymmetry, is defined as:
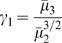
where *µ_i̅_* is the i-*th* central moment of the distribution [26].

#### Firing rate power spectrum

The power spectral density of the network firing rate obtained with a bin width of 10 ms was computed by using Welch's averaged modified periodogram method (*pwelch* function in Matlab) after a low pass filtering with a cut-off frequency of 25 Hz to prevent aliasing.

#### Network correlation coefficient

For each pair (i,j) of neurons the correlation coefficient [26] ρ_i,j_

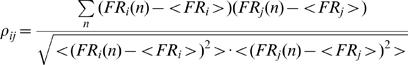
of the neuron firing rates *FR(n)* was computed in a bin width varying from 20 to 1000 ms. ρ_i,j_ was then averaged over all pairs to obtain the *network correlation coefficient* ρ̅.

#### Burst definitions

As there is no standard definition of *burst*, we considered three alternative definitions.

*Definition 1* : The distribution of inter-spike intervals (ISI) between successive spikes in the network was computed and fitted by a bi-exponential function:
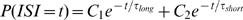
where C_1_ and C_2_ are two constants and τ*_short_* and τ*_long_* are a fast and a slow time constant, respectively. The network firing rate with a bin width equal to τ*_long_* was calculated and strips of adjacent bins, each counting more than two spikes, were considered as bursts.

*Definition 2*: a burst was identified as an ensemble of consecutive spikes separated by a time interval smaller than the average inter-spike interval (*ISI̅* ). *ISI̅* varied from 2 to 20 ms for hippocampal networks and from 10 to 50 ms for leech networks.

*Definition 3:* the network firing rate with a bin width equal to *ISI̅* was calculated, as well as its average value. A burst was identified in the network firing rate as a strip of adjacent bins in which each bin has a number of spikes higher than the average firing rate (see [27], [28]).

For the three burst definitions, only bursts containing more than three spikes were considered. The *burst size* is the total number of spikes within the burst, and its *duration* is the time interval between the first and the last spike of the burst.
